# Modelling the metabolism of protein secretion through the Tat route in *Streptomyces lividans*

**DOI:** 10.1186/s12866-018-1199-3

**Published:** 2018-06-14

**Authors:** José R. Valverde, Sonia Gullón, Rafael P. Mellado

**Affiliations:** 1 Scientific Computing Service. Centro Nacional de Biotecnología (CNB-CSIC), Madrid, Spain; 2 Departamento de Biotecnología Microbiana. Centro Nacional de Biotecnología (CNB-CSIC), Madrid, Spain

**Keywords:** Biotechnology, Metabolic engineering, Genome-scale metabolic network, FBA modelling, Streptomyces lividans, Tat-dependent protein secretion, Sec-dependent protein secretion

## Abstract

**Background:**

*Streptomyces lividans* has demonstrated its value as an efficient host for protein production due to its ability to secrete functional proteins directly to the media. Secretory proteins that use the major Sec route need to be properly folded outside the cell, whereas secretory proteins using the Tat route appear outside the cell correctly folded. This feature makes the Tat system very attractive for the production of natural or engineered Tat secretory proteins. *S. lividans* cells are known to respond differently to overproduction and secretion of Tat versus Sec proteins. Increased understanding of the impact of protein secretion through the Tat route can be obtained by a deeper analysis of the metabolic impact associated with protein production, and its dependence on protein origin, composition, secretion mechanisms, growth phases and nutrients. Flux Balance Analysis of Genome-Scale Metabolic Network models provides a theoretical framework to investigate cell metabolism under different constraints.

**Results:**

We have built new models for various *S. lividans* strains to better understand the mechanisms associated with overproduction of proteins secreted through the Tat route. We compare models of an *S. lividans* Tat-dependent agarase overproducing strain with those of the *S. lividans* wild-type, an *S. lividans* strain carrying the multi-copy plasmid vector and an α-amylase Sec-dependent overproducing strain. Using updated genomic, transcriptomic and experimental data we could extend existing *S. lividans* models and produce a new model which produces improved results largely extending the coverage of *S. lividans* strains, the number of genes and reactions being considered, the predictive behaviour and the dependence on specification of exchange constraints. Comparison of the optimized solutions obtained highlights numerous changes between Tat- and Sec-dependent protein secreting strains affecting the metabolism of carbon, amino acids, nucleotides, lipids and cofactors, and variability analysis predicts a large potential for protein overproduction.

**Conclusions:**

This work provides a detailed look to metabolic changes associated to Tat-dependent protein secretion reproducing experimental observations and identifying changes that are specific to each secretory route, presenting a novel, improved, more accurate and strain-independent model of *S. lividans*, thus opening the way for enhanced metabolic engineering of protein overproduction in *S. lividans*.

**Electronic supplementary material:**

The online version of this article (10.1186/s12866-018-1199-3) contains supplementary material, which is available to authorized users.

## Background

*Streptomyces* are non-pathogenic gram-positive soil bacteria, members of the *Actinobacteria* phylum, displaying mycelial growth, and involved in the breakdown of soil material. They are well known for their ability to synthesize antibiotics and other compounds of biotechnological interest, as well as to produce large quantities of extracellular proteins. This last characteristic, coupled to a relatively low endogenous protease activity, makes them attractive for the production of extracellular enzymes of industrial application [1].

The genomes of key *Streptomyces* (*S*.) strains have been compared in detail, and in some cases, completely sequenced [2–4]. A recent genome comparison of 31 known *Streptomyces* genomes found *S. coelicolor* to be the most similar to *S. lividans* [5]. Hybridizations of the genomes of various strains of *S. lividans* (66, TK21, TK24) among themselves and with those of *S. coelicolor* A3(2) and M145, have shown a considerable genome plasticity, accommodating large deletions and extensive amplifications, often linked to conjugative elements such as SLP1 or SLP3 [6–8].

Streptomycetes have a linear genome of approximately 6–12 Mb with a strong G + C bias (~ 72–75%) [9, 10]. Among streptomycetes, *S. lividans* is a preferred host for protein production because it may be efficiently transformed, due to a relaxed exogenous deoxyreibonucleic acid (DNA) restriction system, facilitating the use of functional plasmids and propagation of heterologous DNA sequences [1, 5].

Bacterial protein production studies have shown that secretory proteins are efficiently secreted when overproduced in *S. lividans* [1, 11]. Protein secretion in *S. lividans* makes use of two pathways: the twin-arginine translocation (Tat) route, which secretes folded proteins using proton motive force (PMF) or ΔpH, and the ATP-dependent major secretion (Sec) route, which exports unfolded proteins that are to be folded outside the cell [12]. The 6 kDa Early secretory antigenic target (ESAT-6) secretion system 1 (ESX-1), a type VII secretion system (T7SS) route, has been identified in *S. lividans* but its importance is uncertain [13]. The cost of protein secretion via the Tat route is difficult to measure: it has been suggested that it might require up to 80.000 protons (H^+^) -equivalent to 10^4^ molecules of ATP- per protein as estimated in thylakoid Tat systems in vitro although it might not require a ΔpH in vivo [14]. The energetic cost of secretion through the Sec route has been variously estimated in *E. coli* as 500 ATP for each translocated 25 kDa polypeptide [14], 1 ATP per each 35–40 amino acids (a.a.) [15], 1 ATP per 25 a.a [16]. or 1 ATP per ~ 5 kDa [17], with PMF being able to provide additional driving force when *Sec*A is not bound or in later stages [18]. Although its cost may be higher, the ability to export proteins already folded makes the Tat route very attractive for its potential use in the overproduction and secretion of specific proteins with industrial interest. Previous work has reported the existence of relevant differences in the cellular response to Sec- and Tat-dependent protein secretion [19]. Hence, existing studies based on Sec-mediated protein secretion are not directly applicable to the Tat route.

Genome-scale metabolic networks (GSMNs) together with Flux Balance Analysis (FBA) and the related Flux Variability Analysis (FVA) and Minimization of the Total Flux (MTF) methods, have been used to get a better understanding of the underlying metabolic effects associated with protein production [20–23]. Assuming that metabolic steps are faster than cellular growth and environmental changes, these methods can treat metabolic fluxes as quasi-steady state and compute a range of optimal fluxes of intermediate metabolites under given constraints. The quality of the results will primarily depend on the extension of the metabolic coverage of the model and, secondarily on the quantity and quality of the reference data (expressed as forced flux limits) used to cover up for model shortcomings. Typically, as models become more comprehensive, they require coercion of less additional flux limits and produce more accurate results. The resulting flux distributions should describe the optimal response of a cell within the limits chosen, allowing the description of experimental results and the prediction of adaptive changes, maximum allowable metabolic yields and fluxes, and potential routes for optimization [20, 21]. Understanding the underlying metabolic mechanisms facilitates the identification of potential bottlenecks and targets for gene or gene-expression modification to modulate the yield of desired products [21].

To date, there are no metabolic models to study the impact of protein secretion using the Tat route. In this work we set out to develop such a model and use it to study the differential aspects of metabolic response to protein secretion through the Tat route, specifically comparing *S. lividans* strains overproducing a model Tat protein (agarase) with the *S. lividans* wild type strain, a *S. lividans* strain carrying the multicopy vector plasmid and an *S. lividans* strain overproducing a model Sec protein (α-amylase).

To model Tat-dependent protein secretion, we use experimental growth and secretion data from the overexpression in *S. lividans* TK21 of the *S. coelicolor dag*A gene encoding agarase propagated in the multicopy plasmid pIJ486. *S.lividans* TK21 was selected because it is a non-plasmid derivative of *S. lividans* 66, and has demonstrated efficient secretion under diverse conditions in our hands [11, 12, 19]. Overexpression of the *S. lividans* TK21 α-amylase encoding gene propagated in the same multicopy plasmid pIJ486 was used to compare Tat- to Sec-dependent protein secretion. The use of the same strain and multicopy vector to propagate genes which are phylogenetically very close to those of the host strain, and the comparison with the wild-type and the multicopy vector-carrying strains allowed us to reduce potential biases in the comparisons.

There are, however, no GSMNs to model overproduction of either agarase or α-amylase in *S. lividans* TK21. To model these, the most efficient approach is to start from existing models of related organisms and extend them to account for known genetic differences. The closest existing model corresponds to the production of mouse Tumor Necrosis Factor α (mTNF-α) in *S. lividans* TK24 growing on a minimal medium [22–24]. This model contains 705 reactions and 496 metabolites and was derived from an earlier model for *S. coelicolor* A3(2) developed by Borodina et al. (iIB711) [25]. There are other *S. lividans* models that have been used to explore the production of cellulase A through ^13^C-based metabolic flux analysis (71 reactions, 35 metabolites) [26] and of xyamenmycin (82 reactions, 86 metabolites) [27], but they are much less detailed, and there is another, iIB711-derived, model published on the web by the SurreyFBA group [28]. A novel model for *S. coelicolor* (iMK1208) [29] might also serve as the basis for a new, better model for *S. lividans*. The availability of these models, detailed genome comparison studies and the complete sequences of *S. lividans* TK24 and *S. lividans* 66, facilitates the design of new models adapted to the production of agarase and α-amylase by *S. lividans* TK21. Additionally, there is information available on amino acid uptake rates during heterologous protein production in *S. lividans* TK24 that could be used as reference for adjusting other FBA models [22].

The closeness of our production systems to existing models makes them especially attractive as a starting point. However, given the large genome plasticity of *Streptomyces*, and the access to new data not available at the time of their design, existing models should be thoroughly reviewed and adapted to ensure that they match all the novel information currently available. In this manuscript, we analyse the metabolic impact of Tat-mediated agarase secretion on *S. lividans* TK21 developing new GSMN models.

## Methods

### Bacterial strains and culture medium

*S. lividans* TK21, a non-plasmid derivative from *S. lividans* 66 (John Innes Center Collection, Norwich UK) was a generous gift from D. A. Hopwood and was used as the wild type strain [30]. Overproduction of agarase and α-amylase was achieved using multicopy plasmids carrying the corresponding genes. *S. lividans* TK21 (pAMI11) and *S. lividans* TK21 (pAGAs5) contain plasmids pAMI11 and pAGAs5 respectively. Plasmid pAMI11 [31] and pAGAs5 are pIJ486 [32] derivative multicopy propagated plasmids carrying the *S. lividans* α-amylase encoding gene (*aml*B) and the *S. coelicolor* agarase gene (*dag*A) under the control of their own promoters, respectively [19, 33].

Mannitol was used as carbon source since glucose has been shown to negatively affect agarase secretion [33, 34]. Mycelia stored at − 80 °C were cultured in flasks of 25 ml with 5 ml of yeast extract-malt extract (YEME) liquid medium with kanamycin at 10 μg·ml^− 1^ final concentration at 30 °C and 250 rpm (rpm). After 72 h of incubation 0,5 ml of the first pre-cultures were grown in 25 ml flasks for 24 h under the same conditions. After that, the second pre-cultures were centrifugated and biomass collected and used to inoculate cultures at an initial concentration of 0.1 g (wet weight) per L. Bacterial cells were grown in 400 ml of minimal liquid medium (NMMP): 1% mannitol, 2 g/L (NH_4_)_2_ SO_4_, 5 g/L Bacto™casamino acids, 0.6 g/L MgSO_4_·7· H_2_O, 150 ml/L of 0.1 M Na H_2_PO_4_/K_2_HPO_4_ and 1 ml/L minor elements solution (containing 1 g/L ZnSO_4_·7 H_2_O, 1 g/L FeSO_4_·7H_2_O, 1 g/L MnCl_2_·4 H_2_O and 1 g/L anhydrous CaCl_2_), and were incubated in 2 L regular flasks at 30 °C and 250 rpm. Biomass concentration was determined using the cell dry weight (DW). Measurements were performed in triplicate.

### Enzyme activities

To determine extracellular agarase and α-amylase activities, samples were taken from the supernatants of the different bacterial cell cultures at each time and proteins present in the samples were concentrated by precipitation with ammonium sulphate brought to 80% saturation; the precipitated protein was collected by centrifugation at 13,000 g for 30 min. and dissolved in 20 mM phosphate buffer (pH 7.0) for α-amylase and in 50 mM imidazole-HCl (pH 6.5) for agarase.

The amount of agarase and α-amylase (mmol) was calculated using purified agarase [35] and commercial α-amylase from *Bacillus amyloliquefaciens* (Sigma ref. A7595) as references to perform standard titration curves at different known concentrations. The protein concentration in the various samples was determined using the BCA protein assay kit (Pierce), as indicated by the supplier. Activities were determined as previously described [33, 36] using supernatants from three independent cultures grown under identical conditions.

### Metabolic models

Initial models for wild-type *S. lividans* TK21 were based on published data for *S. lividans* TK24 [24], which will be hereinafter referred to as iIL708, and on the model iMK1208 published by Kim et al. for *S. coelicolor*A3(2) [29]. The iIL708 model was reconstructed from the published data and verified to reproduce the original results.

The existing models were updated to account for new information not available at the time of their respective publication. The genome sequences of the *S. coelicolor* and *S. lividans* strains were retrieved from the European Nucleotide Archive (ENA), and compared against each other at the coding sequence level using Blast-based RATT [37] to verify and complete the annotation, match gene identifiers, confirm missing genes, and search for isozymes that could provide functional alternatives to missing genes.

The newly generated models for the wild type *S. lividans* TK21 were subsequently modified to include reactions for the pIJ486-carrying strain and for strains producing agarase or α-amylase following a procedure based on that of Lule et al. [24], using a plasmid copy number Pn = 100 and an efficiency Pc = 4000, and defining a lump reaction for messenger RNA (mRNA) transcription and translation into protein with several modifications. We used an estimated cost of 4 high-energy bonds per amino acid and 2 per mRNA nucleotide (nt), expressed as ATP, and an mRNA yield of 30 [38] or 4000 (see below) proteins per mRNA.

We also modified the protein production lump reaction so that the ADP produced is the result of energy consumption, that phosphate (P_i_) also includes the P_i_ released when NTPs are incorporated into mRNA (2 P_i_ per nt), and that the mRNA is included as a product. We have used more precise data for mRNA and energy consumption needs in the case of agarase and α-amylase. Two mRNA degradation lump reactions for each mRNA were added to allow the cell to recover the nucleotides used in mRNA production after mRNA decay either through hydrolysis to NMPs or through phosphorylation to NDPs. Secretion via the Tat route was modelled as an export reaction since the cost of the PMF or of ΔpH is not well characterized [14], while secretion through the Sec route was added as a separate ATP-dependent reaction. Additional exchange reactions for each recombinant protein were added as well.

We have also built models using a cost of 8·10^4^ H^+^ / protein for Tat secretion and compared the results with the default model.

### Constrained-based modelling

We used both the Matlab-based OpenCobra toolkit [39] and the R-based Sybil package [40] to run FBA, MTF and FVA calculations on each of the models. Initial constraints used were derived from experimental data for biomass, agarase and α-amylase and from known uptake rates for heterologous protein production in *S. lividans* TK24 [22]. The wild-type and the derived strains were modelled using mannitol as the main carbon source. Additional models employing reduced (using only the lower bounds for mannitol and amino acid exchange) or minimal (limiting only biomass and protein production) exchange rate constraints were also tested to allow for strain differences and to test the predictive power of the model.

The optimal flux distributions computed using the MTF were compared using the Kolmogorov-Smirnov and Wilcoxon tests on flux values and on normalized vector differences (δƒ) of the active metabolic networks (AMN), defined as the set of reactions that were active in any of the strains being compared, using the R statistical package.

## Results

### Biomass and protein production quantification

**Fig. 1:**
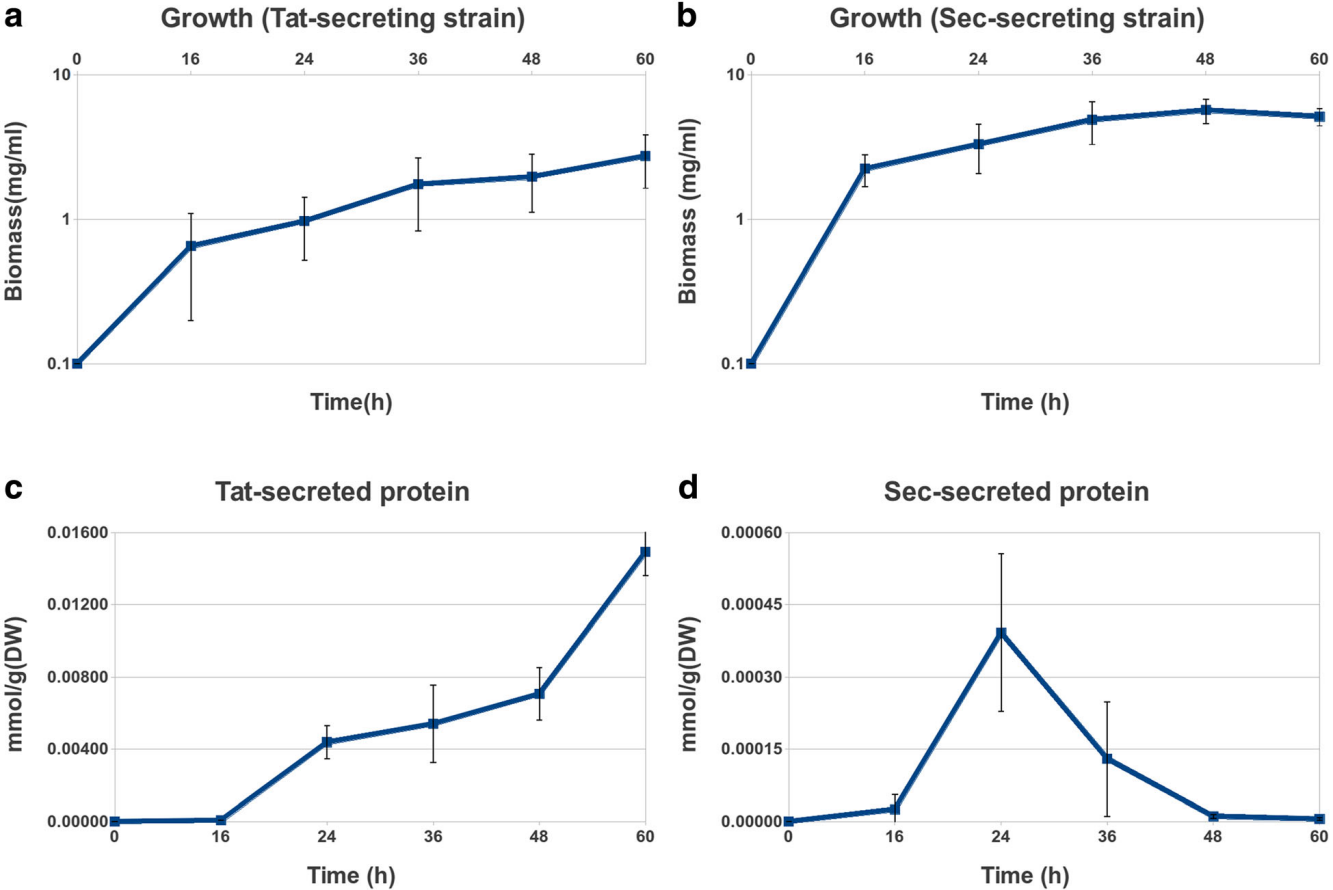
Experimental growth and secretion curves. Logarithmic growth curves of the *S. lividans* TK21 strain secreting agarase using the Tat route and of the *S. lividans* TK21 strain secreting α-amylase are shown in panels **a** and **b** respectively and are expressed in grams of dry weight mass per liter. Agarase secreted through the Tat route and α-amylase secreted through the Sec route as a function of time are shown in panels **c** and **d** respectively and are expressed in milli-mols per gram of dry weight. Time is expressed in hours in all cases. Bars show the standard deviation

Experimental measurements are shown in Fig. 1 and are available as an additional file (see Additional file 1) (experimental growth and secretion data) and show that each protein displays a different temporal secretion pattern. There may be some Sec-dependent agarase secretion during the exponential growth phase, until the switch to Tat-dependent production of extracellular agarase occurs at about 24 h, which reaches a maximum at 60 h. In the case of α-amylase, measured extracellular protein production is maximal at 24 h, corresponding to late exponential growth, and decreases afterwards [19, 41].

To simplify comparisons and maximize differences, we have chosen to analyse the models at the time points of maximal protein secretion in each case: for agarase, 60 h (biomass 0.065 g_DW_/h and protein secretion 6.552·10^− 4^ mmol/g_DW_/h), and for α-amylase 24 h (biomass 0.135 g_DW_/h and protein secretion 4.5887·10^− 5^ mmol/g_DW_/h).

### Metabolic models

We have revised the existing *S. lividans* models based on iIB711 to integrate the newly available experimental and genome sequence data. As of today, the largest *S. lividans* models are iIL708 and a web-published model from SurreyFBA. A comparison of the models shows that SurreyFBA differs from iIB711 only in the removal of nine TK24 genes deemed missing, while iIL708 removes only four genes and modifies various reactions, including 4 reactions not present in the SurreyFBA model and one not present in iIB711. Additional sequence-level comparisons of the various genomes available for *S. coelicolor* and *S. lividans* allowed us to further correct the model identifying additional isozymes for deleted or previously considered missing genes. Since iIL708 does not contain gene information, we only considered the metabolic reactions in its derivative model.

We have also investigated models of *S. coelicolor* more recent than iIB711 that might serve as a foundation for improved *S. lividans* models. We have chosen iMK1208 which incorporates numerous enhancements as the basis for a new *S. lividans* TK21 model. References to *S. coelicolor* genes missing in *S. lividans* were removed together with the corresponding reaction whenever no alternative enzyme could be identified in *S. lividans* genomes. For comparative purposes, we have conserved the *S. coelicolor* gene name until an annotated genome for TK21 providing definitive names is available.

The only known difference of metabolic interest between the TK24 and TK21 strains corresponds to gene *SCO0984* which encodes 3-hydroxybutyryl-CoA dehydrogenase (EC 1.1.1.157) in *S. coelicolor*. *S. lividans* TK24 and *S. lividans* 66 contain alternative genes that can provide for the same function. Since these other genes have also been identified in *S. lividans* TK21, it seems reasonable to assume that the function of *SCO0984* is also present in TK21. Since the model derived from iIL708 lacks gene information and the model derived from iMK1028 does not make use of that gene, it should be possible to apply the new models without modifications to *S. lividans* TK21, *S. lividans* TK24 and *S. lividans* 66.

In order to study the impact of protein overproduction and secretion through the Tat route, we added support for the use of pIJ486 as a vector for heterologous protein production and suitable reactions to model agarase production and secretion. To better understand the aspects of protein secretion in *S. lividans* TK21 that are specific of the Tat route, we also modelled production of α-amylase and its secretion through the Sec route using data obtained in *S. lividans* TK21 for comparison.

To estimate protein production costs, the sequences for agarase and α-amylase were retrieved from the EMBL database (entries X05811 and Z85949) and used to derive protein and mRNA composition. The sequence for the agarase encoding gene (*dag*A) documents four promoters that are functional in *S. lividans* TK21 [42]. Although these promoters may not be equally effective, we used them to calculate an average metabolic cost of *dag*A mRNA transcription. The mRNA from *amlB* is known to be at least 1925 nt long that were used to estimate transcription costs. Translation costs were calculated for their corresponding pre-proteins including their signal peptides, 309 a.a. for agarase and 573 a.a. for α-amylase.

As a result of these modifications, we have constructed two new models for the simulation of protein production by *S. lividans*. Model iJV710 derives from iIL708 and model iJV1220 is based on iMK1208 modified for *S. lividans* and protein secretion. Both new models include additional support for plasmid propagation, protein production, mRNA degradation and overproduction of agarase secreted through the Tat route and of α-amylase secreted through the Sec route.

Both models are deemed suitable for modelling either the *S. lividans* TK21, *S. lividans* TK24 or *S. lividans* 66 strains, whether wild-type, plasmid-carrying or extracellular protein-producing. All the models generated have been tested to be compatible with the OpenCobra Toolkit and the R Sybil package and are provided separately (Additional file 2) (GSMN models). The main differences between the models are summarized in Table 1.

**Table 1:** Summary comparison of the models designed for *S. lividans*

	iJV710	iJV1220
Number of genes	0 (710)^a^	1220^b^
Number of reactions	713	1446
Number of metabolites	502	1867
Mass balanced	No	Yes
Amylase	Yes	Yes
Agarase	Yes	Yes
mRNA decay	No	Yes
Branched-chain fatty acids	No	Yes
Menaquinone biosynthesis	No	Yes
Updated biomass equation	No	Yes
Updated energy parameters	No	Yes

^a^The model contains no gene information, 710 genes are assumed since it is based on iIL708 and adds reactions corresponding to 2 new genes

^b^iJV1220 is based on iMK1208, removing 4 genes, and adding genes for *aml*B, *dag*A, RNAse, PNPase and the secretion complexes

### Modelling results

We used model iJV710 to compare the wild-type and plasmid-carrying strains with those producing Tat-secreted agarase and Sec-secreted α-amylase using mannitol as carbon source. To model mannitol consumption, the uptake of glucose was set to zero, the flux of reaction R147 (ATP + GLC → ADP + G6P) which had been coerced in iIL708 to force all glucose consumption to follow this route, was freed to use bounds 0–1000, and the uptake of mannitol was set to the reference value of glucose uptake. The results of the FBA calculations are included as a separate file (Additional file 3) (MTF and FVA results for iJV710) and relevant changes are summarised in Table 2 and detailed in (Additional file 4).

**Table 2:** Summary of changes in the Tat-secreting strain observed using model iJV710

Pathway	Tat-secreting vs. Sec-secreting				Tat-secreting vs. Plasmid-bearing			
	N	N_AMN_	P _alt = Tat < Sec_	P _alt = Tat > Sec_	N	N_AMN_	P_alt = Tat < pIJ486_	P_alt = Tat > piJ486_
Carbon sources	138	26	0.540	0.021	138	29	0.110	0.733
Sulphate metabolism	5	4	0.105	1	5	4	0.779	0.105
Glycolysis	23	13	0.0004	0.9259	23	13	0.002	0.926
TCA cycle	34	18	0.233	0.777	34	19	0.289	0.721
PPP	16	9	0.046	0.962	16	9	0.0002	0.999
Anaplerosis	13	5	0.147	0.896	13	5	0.264	0.799
Energy metabolism	9	6	0.409	0.650	9	6	0.591	0.469
Amino acid biosynthesis	90	56	0.168	0.417	90	64	0.0001	1
Nucleotide biosynthesis	81	45	0.00014	0.978	81	46	0.0004	0.916
Lipid biosynthesis	49	36	0.0001	1	49	37	0.0004	0.973
Cofactor biosynthesis	54	41	0	0.976	54	42	0	0.976
Macromol. biosynthesis	10	10	0.095	0.917	10	10	0.109	0.905

Comparisons of the Tat-secreting strain using the model iJV710 based on MTF analysis of the AMN with pathways grouped in broad subsystems. N is the number of reactions in the subsystem, N_AMN_ is the number of subsystem reactions that are active in either of the two strains being compared, P is the *P* value obtained using the specified alternative hypothesis (i.e. when *P* < 0.05 the specified alternative hypothesis cannot be rejected). Only subsystems with N_AMN_ > 4 are reported

*Abbreviations*: *TCA cycle* tricaboxylic acid (Krebs) cycle, *PPP* pentose phosphate pathway

Before comparing Sec- and Tat-dependent results, we checked the relative impact of plasmid expression and of changing the carbon source to define their relative contribution to the changes observed. A detailed comparison of the wild-type and plasmid-carrying strains grown with glucose or mannitol, shows only minor nutrient-related differences, while forcing production of the plasmid at 100 copies per cell has a relatively small impact (likely due to the small proportion of plasmid and marker protein produced). The overall differences between the distributions of the wild-type and plasmid-carrying strain were not statistically significant (See Additional file 4).

Global comparison of the flux distributions showed that Tat-dependent protein secretion was statistically significant with respect to the Sec-secreting, plasmid-carrying and wild-type strains (*P* < 0.05). After a detailed analysis of the differences, besides the effects due to mannitol usage and plasmid production, we identified differences between the protein producing and the reference plasmid-carrying strains that affect the usage of amino acids, reflected both in the uptake and metabolic (catabolism and biosynthesis) rates, carbon metabolism (due to the substitution of glucose by mannitol, but also affecting glycolysis and the citrate and pentose phosphate routes), energy metabolism, nucleotide, and metabolite transport. Many of these changes showed a differential behaviour depending on whether the strain was using the Tat (agarase) or the Sec (α-amylase) secretion route: synthesis of macromolecules, cofactors, fatty acids, nucleotides, amino acids, energy production and glycolysis was generally smaller in the Tat-secreting, agarase producing, strain (Additional file 4).

The iJV1220 model provides a more complete view of the metabolism including additional and important routes. The simulation results obtained with iJV1220 are provided separately (Additional file 5) (MTF and FVA results for iJV1220) and broadly summarised in Table 3 and detailed in (Additional file 6). By including numerous additional reactions, the iJV1220 model also permits the identification of additional, previously not considered, fluxes, such as secondary metabolism, ion transport and exchange rates.

**Table 3:** summary of changes observed in the Tat-secreting strain using model iJV1220

Subsystem	Tat-secreting vs. Sec-secreting				Tat-secreting vs. Plasmid-bearing			
	N	N_AMN_	P _alt = Tat < Sec_	P _alt = Tat > Sec_	N	N_AMN_	P_alt = Tat < pIJ486_	P_alt = Tat > pIJ486_
Amino acid metabolism	187	81	0.228	0.773	187	82	0.044	0.956
Carbon metabolism	131	17	0.333	0.679	131	18	0.419	0.594
Cell envelope	577	292	0.000	1.000	577	292	0.000	1.000
TCA	17	11	0.179	0.838	17	10	0.153	0.864
Cofactor biosynthesis	214	148	0.000	1.000	214	148	0.000	1.000
Exchange	216	43	0.646	0.357	216	45	0.578	0.425
Glycolysis and gluconeogenesis	21	13	0.185	0.829	21	12	0.397	0.625
Inorganic Ion Transport and Metabolism	59	15	0.270	0.744	59	15	0.230	0.782
Nucleotide metabolism	125	47	0.023	0.978	125	47	0.023	0.978
Oxidative Phosphorylation	17	7	0.185	0.847	17	6	0.188	0.852
PPP	15	10	0.741	0.285	15	8	0.604	0.437
Transport, Membrane	167	22	0.500	0.509	167	25	0.431	0.577
Unassigned	16	4	0.500	0.614	16	4	0.500	0.614
Amylase secretion	5	4	0.009	0.996	5	0	NA	NA
Agarase secretion	5	4	0.996	0.009	5	4	0.996	0.009

Comparisons of the Tat-secreting strain using the model iJV1220 based on MTF analysis of the AMN with pathways grouped in broad subsystems. N is the number of reactions in the subsystem, N_AMN_ is the number of subsystem reactions that are active in either of the two strains being compared, P is the P value obtained using the specified alternative hypothesis (i.e. when *P* < 0.05 the specified alternative hypothesis cannot be rejected). Only subsystems with N_AMN_ > 4 are reported

*Abbreviations*: *TCA cycle* tricaboxylic acid (Krebs) cycle, *PPP* pentose phosphate pathway, *NA* not applicable

Global differences among the AMN flux distributions are confirmed when using the iJV1220 model, with increased statistical significance (probability *P* = 2.2·10^− 16^). Detailed inspection of the individual reactions (Additional file 6) provides additional information regarding the differences among the Tat- and Sec-secreting strains: the Tat-secreting, agarase producing, strain shows reductions in the flux of specific reactions in the cell envelope, glycolysis, oxidative phosphorylation, cofactor biosynthesis, methionine metabolism and nucleotide metabolism with an unbalance in nucleotide diphosphate kinase (SCO2612) towards increased production of NTPs and reduced production of dNTPs, and increased fluxes in the pentose phosphate pathway (PPP) and the metabolism of several amino acids.

Previous models containing less reactions required supplementary experimental information provided as additional flux limits to produce sensible results, and ignored metabolite exchange fluxes that had not been explicitly measured experimentally [24].

We checked the dependency of the model on the specification of metabolite exchange fluxes by loosening the flux limits: we run calculations using relaxed (defining only the lower bounds on mannitol and amino acid exchange) or minimal (allowing free exchange of any metabolite and limiting only biomass and minimal protein production) constraints. Using these less-constrained models, we could monitor the uptake and excretion rates of numerous metabolites whose exchange could not be considered in previous simulations (see Additional file 5, MTF and FVA results for iJV1220). The results agree with observations from growth in minimal medium: besides numerous ions not considered in previous models, the model identified amino acids as the preferred nutrients, in agreement with experimental observations (when grown with casamino acids, amino acids are the preferred carbon sources, and as they start to diminish, the cells start using other carbon sources [19, 22, 43]). Overall, the computed MTF fluxes and their respective FVA limits were remarkably similar irrespective of whether they were computed with extensive experimental constraints, with relaxed or even with no constrains at all (other than biomass and minimal secreted protein production) (See Additional file 5, MTF and FVA results for iJV1220) and agreed with experimentally observed exchange rates, which were within the predicted FVA limits.

We have also used model iJV1220 to explore the potential impact of using a secretion cost as high as that proposed for in vitro chloroplast thylakoid systems by setting the cost to 8·10^4^ H^+^ per protein. The results (provided as Additional file 7) indicate that the theoretical maximum secretion of heterologous protein is not affected, and the associated flux changes may be interpreted as leading to maintain the pool of free H^+^ (increased glycolysis and associated pathways, pyruvate metabolism, nucleotide salvage, membrane transport, and decrease of oxidative phosphorylation, PPP, TCA -which produce NADPH reducing the H^+^ pool- and exchange reactions). Simulations using 10^4^ ATP (data not shown) led to a different flux distribution showing that the two costs are not metabolically equivalent.

## Discussion

In this work we describe the utilization of metabolic models to describe the experimental growth and secretion rates of Tat-secreted agarase and Sec-secreted α-amylase overproduced in *S. lividans* TK21. Our experimental measures confirm previous observations [19, 33–35, 42]. To facilitate identification of the effects due to protein production, we restrict comparisons to maximal production phases using the same medium, host strain and vector.

We present here two new metabolic models, iJV710, which may be used to obtain comparisons with previously published data, and iJV1220 which largely extends existing *S. lividans* models. Previous models for *S. lividans* TK24 were adapted from iIB711 for *S. coelicolor*, using hybridization comparison data [6–8]. Due to the large genome plasticity of streptomycetes, we updated this model using newly published data, most notably the genome sequences of *S. lividans* TK24 and *S. lividans* 66 [2, 3], to identify any potential changes specific to *S. lividans* TK21. Our comparative analyses suggest that, although we were initially interested in modelling *S. lividans* TK21, and according to available information at the time of writing, our metabolic models should also be valid at least for *S. lividans* 66 and *S. lividans* TK24 (except that iJV1220 uses *S. coelicolor* gene names). We extended our models to add plasmid propagation and protein production reactions. Thus, the same model may be used to simulate the wild-type, plasmid-carrying and protein producing strains by simply setting the flux limits of the corresponding reactions to appropriate values.

Although we have used the best data available to maximize model accuracy, we still had to approximate some reaction costs: plasmid and indicator protein production is approximate since the plasmid sequence is not available; although the translation process consumes actually both ATP and GTP as energy sources, the cost of protein translation was expressed in terms of summarized high-energy phosphate bonds of ATP for simplicity, considering them metabolically inter-convertible and following common practice (e.g. [24, 38, 44]); secreted protein and mRNA composition ignores potential leading or trailing sequences or preferences for mRNA isoforms; mRNA decay is much more complex than expressed and is included mainly to allow the cell to recover mRNA nucleotides; and protein secretion cost is based on average estimates (as it is not currently possible to determine it with more precision). The potential effect of PMF or ΔpH in either the Tat or the Sec routes is ill-defined and has not been considered in the current models, pending availability of additional information.

Additional reactions might be included in more detailed models, such as the removal of the signal peptide by signal peptidases, glucose inhibition of Tat-secretion, intracellular protein accumulation and extra- and intra-cellular protein degradation would be needed to model other observed changes in protein secretion, but there is currently not enough information to model these steps properly.

FBA and associated methods have been previously applied to batch culture data (e.g. [44, 45]) in other organisms. While previous *S. lividans* models were derived for fed-batch cultures, we successfully used our models for batch and fed-batch cultures (data not shown), supporting their utility in a broader range of situations.

These models enabled us to explore the potential metabolic costs of Tat-mediated protein secretion in batch cultures using the best data available to date. Overall model predictions agree with experimental observations [23, 24, 26]. The metabolic differences between the plasmid-bearing and wild type strains are small, as is the case between the use of glucose or mannitol as carbon sources. The latter is to be expected since mannitol should readily be converted to D-mannitol-1P and subsequently β-D-fructose-6P, but fails to explain the experimentally observed negative effect of glucose on growth and protein secretion suggesting it may be exerted through non-metabolic mechanisms. While the differences between the wild type, the pIJ486 bearing and the Sec- secreting strains are easy to interpret, it is more difficult to assert the relative importance of the high number of differences observed when comparing Tat (agarase) and Sec (α-amylase) results. Most differences agree with the experimental observations that Tat-mediated protein production is associated to slowed cell growth at all stages. The metabolic predictions identify too the metabolic trends corresponding to differential gene expression data [19]. However, it is difficult to draw clear conclusions: maximum secretion is observed at different growth rates, the production of proteins with different size, composition and yields might be affected by amino acid usage, some gene clusters containing different enzymes may be co-regulated, and alternative isozymes may be subject to differential expression, hence the model may potentially show a behaviour that may seem occasionally inconsistent with expression microarrays, especially since *Streptomyces* spp. has many duplicated genes [6, 8, 45].

The predictions obtained with iJV710 permit direct comparison with those obtained by previous *S. lividans* models and show reduced fluxes in glycolysis, lipid, nucleotide and cofactor metabolism in Tat versus Sec-dependent secretion.

The fluxes computed with the more complete model, iJV1220 show statistically significant changes related to cell envelope, cofactor and nucleotide biosynthesis similarly to iJV710, with differences that can be ascribed to the inclusion of previously unconsidered relevant routes. Being more comprehensive, iJV1220 provides additional details and reproduces better the known experimental behaviour [11, 19, 22, 26, 43]. The iJV1220 model displayed a better predictive behaviour, reproducing microarray expression data [19] and experimental observations of metabolite exchange rates when most constraints on exchange flux limits were relaxed or removed. Since iJV1220 is more comprehensive, produces better results, and should be valid too for *S. lividans* TK24 and *S. lividans* 66, we favour its use. Since it has been shown that *S. lividans* may efficiently produce heterologous proteins with a different codon usage bias [46], the availability of this new, improved metabolic model offers the possibility of using it to study protein production in *S. lividans* with minimal assumptions. Additionally, iJV1220 also adds gene information and, therefore, may be used to explore the potential effect of genetic modifications and to identify potentially interesting target genes controlling protein overproduction and secretion. The upper limits of protein secretion predicted by FVA and by setting maximal protein secretion as the objective suggest that there could be room for increasing heterologous protein production using either the Tat or the Sec route (see Additional files 3 and 5). Future work to improve protein production should exploit comprehensive modelling and address the influence of non-metabolic factors, which are currently difficult to incorporate into FBA models.

## Conclusions

Modeling of Tat-dependent protein secretion identifies a large number of changes with respect to Sec-dependent protein secretion or the plasmid-bearing and wild-type strains, both at the subsystem level and at the level of individual reactions. These changes can be related to observed behaviour and reproduce experimental results. Variability analysis shows that there is ample room for improvement in protein secretion until the protein production limits of the system are reached, opening the possibility of using these models in protein secretion bioengineering of *S. lividans*.

Both, iJV710 and iJV1220 have proven useful to obtain insights into the metabolism associated with wild-type, plasmid-carrying and extracellular protein-production either via the Tat or Sec routes using *S. lividans,* irrespective of the host strain. Since iJV1220 provides more information and has demonstrated to respond well when using relaxed or minimal exchange limits, it provides the best existing option for metabolic modelling in *S. lividans*, especially when limited information is available on potential metabolic constraints. The availability of iJV1220, a more extensive model that may be valid for *S. lividans* TK21, *S. lividans* TK24 and *S. lividans* 66, and supports secretion through the Tat and/or Sec routes, should facilitate future metabolic models of protein secretion, leaving selection of the secretion route or host strain as choices to be decided on a case-by-case basis considering additional factors.

## Additional files

Additional file 1: Experimental growth and secretion data. Excel file containing experimentally determined biomass and protein secretion data for the agarase and α-amylase producing strains. (XLS 11 kb)

Additional file 2: GSMN models. Zip file containing the new models, iJV710 and iJV1220 in SBML format. As shipped, the models represent the wild-type strain but contain support for the plasmid-carrying and various protein overproducer strains. In order to model these other strains, the limits of the corresponding reactions should be set to appropriate values. (ZIP 389 kb)

Additional file 3: MTF and FVA results for iJV710. Excel file containing: the list of metabolites and reactions considered in the model, the results obtained with the model subject to lower and upper bound constraints using reference values and optimizing for (constrained) biomass production, the results obtained releasing the upper bounds on protein production and optimizing for protein secretion (to maximize the effect of protein production and secretion). (XLS 500 kb)

Additional file 4: Comparison tables for iJV710. Tables comparing results obtained using model iJV710 for the protein secreting strains to those of the wild-type and the plasmid carrying strains. (XLS 4108 kb)

Additional file 5: MTF and FVA results for iJV1220. Excel file containing the list of metabolites and reactions in model iJV1220, together with the results of MTF and FVA calculations using reference, relaxed and minimal constraints. Relaxed constraints consist in setting the larger exchange limit to 1000 and the minor exchange limit to the reference value, or to the reference value minus one or two standard deviations. Minimal constraints consist in using no limits for exchange reactions except for biomass production and protein secretion). (XLS 2141 kb)

Additional file 6: Comparison tables for iJV1220. Tables comparing the results obtained using the iJV1220 model for the protein secreting strains with those of the plasmid bearing and wild-type strains. (XLS 11751 kb)

Additional file 7: Comparison tables and MTF results of simulating Tat secretion using a cost of 80,000 protons per protein. (XLS 3191 kb)

## Abbreviations

(NH_4_)_2_ SO_4_: Ammonium sulphate
a.a.: amino acid
ADP: Adenosine diphosphate
amlB: Gene enconding amylase
AMN: Active metabolic network
ATP: Adenosine triphosphate
CaCl_2_: Calcium chloride
dagA: Gene enconding agarase
DNA: Deoxyribonucleic acid
dNTP: deoxynucleoside triphosphate
DW: Dry weight
EC: Enzyme commission
EMBL: European Molecular Biology Laboratory
ENA: European Nucleotide Archive
ESX-1: 6 kDa Early secretory antigenic target (ESAT-6) secretion system 1
FBA: Flux balance analysis
FeSO_4_: Iron(II) sulphate
FVA: Flux variability analysis
GSMN: Genome-scale metabolic network
GTP: Guanosine triphosphate
H^+^: proton
H_2_O: water
HCl: Hydrogen chloride
K_2_HPO_4_: Dipotassium hydrogenphoshpate
MgSO_4_: Magnesium sulphate
MnCl_2_: Manganese(II) chloride
mRNA: messenger RNA
MTF: Minimization of total flux
mTNF-α: mouse tumor necrosis factor α
NA: Not applicable
NADPH: Nicotinamide adenine dinucleotide phosphate (reduced form)
NaH_2_PO_4_: Monosodium dihydrogen orthophosphate
NDP: Nucleoside diphosphate
NMP: Nucleoside monophosphate
nt: nucleotide
NTP: Nucleoside triphosphate
P_i_: Inorganic phosphate
PMF: Proton motive force
PPP: Pentose phosphate pathway
RNA: Ribonucleic acid
rpm: revolutions per minute
*S*.: *Streptomyces*
Sec: Secretion pathway
T7SS: Type VII secretion system
Tat: Twin-arginine translocation pathway
TCA: Tricaboxylic acid
YEME: Yeast extract-malt extract
ZnSO_4_: Zinc sulphate

## References

[1] Anné J, Van Mellaert L (1993). *Streptomyces lividans* as host for heterologous protein production. FEMS Microbiol Lett.

[2] Cruz-Morales P, Vijgenboom E, Iruegas-Bocardo F, Girard G, Yáñez-Guerra LA, Ramos-Aboites HE (2013). The genome sequence of *Streptomyces lividans* 66 reveals a novel tRNA-dependent peptide biosynthetic system within a metal-related genomic island. Genome Biol Evol.

[3] Rückert C, Albersmeier A, Busche T, Jaenicke S, Winkler A, Friðjónsson ÓH (2015). Complete genome sequence of *Streptomyces lividans* TK24. J Biotechnol.

[4] Bentley SD, Chater KF, Cerdeño-Tárraga AM, Challis GL, Thomson NR, James KD (2002). Complete genome sequence of the model actinomycete *Streptomyces coelicolor* A3 (2). Nature.

[5] Wang J, Wang C (2017). Metabolic network model guided engineering ethylmalonyl-CoA pathway to improve ascomycin production in *Streptomyces hygroscopicus var ascomyceticus*. Microbial Cell Fact.

[6] LeBlond P, Redenbach M, Cullum J (1993). Physical map of the *Streptomyces lividans* 66 genome and comparison with that of the related strain *Streptomyces coelicolor* A3 (2). J Bacteriol.

[7] Jayapal KP, Lian W, Glod F, Sherman DH, Hu WS (2007). Comparative genomic hybridizations reveal absence of large *Streptomyces coelicolor* genomic islands in *Streptomyces lividans*. BMC Genomics.

[8] Lewis RA, Laing E, Allenby N, Bucca G, Brenner V, Harrison M (2010). Metabolic and evolutionary insights into the closely-related species *Streptomyces coelicolor* and *Streptomyces lividans* deduced from high-resolution comparative genomic hybridization. BMC Genomics.

[9] Benigni R, Petrov PA, Carere A (1975). Estimate of the genome size by renaturation studies in *Streptomyces*. Appl Microbiol.

[10] Gładek A, Zakrzewska J (1984). Genome size of *Streptomyces*. FEMS Microbiol Lett.

[11] Gullón S, Vicente RL, Valverde JR, Marín S, Mellado RP (2015). Exploring the feasibility of the sec route to secrete proteins using the tat route in *Streptomyces lividans*. Mol Biotechnol.

[12] Mellado RP (2011). Summing up particular features of protein secretion in *Streptomyces lividans*. World J Microbiol Biotechnol.

[13] Anné J, Vrancken K, Van Mellaert L, Van Impe J, Bernaerts K (2014). Protein secretion biotechnology in gram-positive bacteria with special emphasis on *Streptomyces lividans*. Biochim Biophys Acta.

[14] Natale P, Brüser T, Driessen AJ (2008). Sec-and tat-mediated protein secretion across the bacterial cytoplasmic membrane—distinct translocases and mechanisms. Biochim Biophys Acta.

[15] Mergulhão FJM, Summers DK, Monteiro GA (2005). Recombinant protein secretion in *Escherichia coli*. Biotechnol Adv.

[16] Liu JK, O’Brien EJ, Lerman JA, Zengler K, Palsson BO, Feist AM (2014). Reconstruction and modeling protein translocation and compartmentalization in *Escherichia coli* at the genome-scale. BMC Syst Biol.

[17] Collinson I, Corey RA, William JA (2015). Channel crossing: how are proteins shipped across the bacterial plasma membrane?. Phil Trans R Soc B.

[18] Anné J, Maldonado B, Van Impe J, Van Mellaert L, Bernaerts K (2012). Recombinant protein production and streptomycetes. J Biotechnol.

[19] Gullón S, Marín S, Mellado RP (2015). Overproduction of a model sec- and tat-dependent secretory protein elicits different cellular responses in *Streptomyces lividans*. PLoS One.

[20] Varma A, Palsson BO. Metabolic flux balancing: basic concepts, scientific and practical use. Bio/Technology. 1994;12

[21] Orth JD, Thiele I, Palsson BØ (2010). What is flux balance analysis?. Nat Biotechnol.

[22] D’Huys PJ, Lule I, Van Hove S, Vercammen D, Wouters C, Bernaerts K (2011). Amino acid uptake profiling of wild type and recombinant *Streptomyces lividans* TK24 batch fermentations. J Biotechnol.

[23] D’Huys PJ, Lule I, Vercammen D, Anné J, Van Impe JF, Bernaerts K (2012). Genome-scale metabolic flux analysis of *Streptomyces lividans* growing on a complex medium. J Biotechnol.

[24] Lule I, D’Huys PJ, Van Mellaert L, Anné J, Bernaerts K, Van Impe J (2013). Metabolic impact assessment for heterologous protein production in *Streptomyces lividans* based on genome-scale metabolic network modeling. Math Biosci.

[25] Borodina I, Krabben P, Nielsen J (2005). Genome-scale analysis of *Streptomyces coelicolor* A3 (2) metabolism. Genome Res.

[26] Bouvin J, Daniels W, Anné J, Nicolaï B, Bernaerts K (2016). Metabolic fluxes in recombinant *Streptomyces lividans* analyzed with 13 C-based metabolic flux analysis. IFAC-PapersOnLine.

[27] Xu MJ, Chen YC, Xu J, Ao P, Zhu XM (2015). Kinetic model of metabolic network for xiamenmycin biosynthetic optimisation. IET Syst Biol.

[28] Surrey Computational Cell Biology Server. http://sysbio3.fhms.surrey.ac.uk/. Accessed 16 Jan 2017.

[29] Kim M, Sang Yi J, Kim J, Kim JN, Kim MW, Kim BG (2014). Reconstruction of a high-quality metabolic model enables the identification of gene overexpression targets for enhanced antibiotic production in *Streptomyces coelicolor* A3 (2). Biotechnol J.

[30] Hopwood DA, Kieser T, Wright HM, Bibb MJ (1983). Plasmids, recombination and chromosome mapping in *Streptomyces lividans* 66. Microbiology.

[31] Palomino C, Mellado RP (2008). Influence of a *Streptomyces lividans* SecG functional analogue on protein secretion. Int Microbiol.

[32] Ward JM, Janssen GR, Kieser T, Bibb MJ, Buttner MJ, Bibb MJ (1986). Construction and characterisation of a series of multi-copy promoter-probe plasmid vectors for *Streptomyces* using the aminoglycoside phosphotransferase gene from Tn5 as indicator. Mol Gen Genet MGG.

[33] Parro V, Mellado RP (1994). Effect of glucose on agarase overproduction by *Streptomyces*. Gene.

[34] Parro V, Mellado RP, Harwood CR (1998). Effects of phosphate limitation on agarase production by *Streptomyces lividans* TK21. FEMS Microbiol Lett.

[35] Parro V, Vives C, Godia F, Mellado RP (1997). Overproduction and purification of an agarase of bacterial origin. J Biotechnol.

[36] Gullon S, Vicente RL, Mellado RP (2012). A novel two-component system involved in secretion stress response in *Streptomyces lividans*. PLoS One.

[37] Otto TD, Dillon GP, Degrave WS, Berriman M (2011). RATT: rapid annotation transfer tool. Nucleic Acids Res.

[38] Kaleta C, Schäuble S, Rinas U, Schuster S (2013). Metabolic costs of amino acid and protein production in *Escherichia coli*. Biotechnol J.

[39] Schellenberger J, Que R, Fleming RM, Thiele I, Orth JD, Feist AM (2011). Quantitative prediction of cellular metabolism with constraint-based models: the COBRA toolbox v2. 0. Nat Protoc.

[40] Gelius-Dietrich G, Desouki AA, Fritzemeier CJ, Lercher MJ (2013). Sybil–efficient constraint-based modelling in R. BMC Syst Biol.

[41] Palacín A, de la Fuente R, Valle I, Rivas LA, Mellado RP (2003). *Streptomyces lividans* contains a minimal functional signal recognition particle that is involved in protein secretion. Microbiology.

[42] Parro V, Mellado RP (1993). Heterologous recognition in vivo of promoter sequences from the *Streptomyces coelicolor* dagA gene. FEMS Microbiol Lett.

[43] Gabarró M, Gullón S, Vicente RL, Caminal G, Mellado RP, López-Santín JA (2017). *Streptomyces lividans* SipY deficient strain as a host for protein production: standardization of operational alternatives for model proteins. J Chem Technol Biotechnol.

[44] Özkan P, Sariyar B, Ütkür FÖ, Akman U, Hortaçsu A (2005). Metabolic flux analysis of recombinant protein overproduction in *Escherichia coli*. Biochem Eng J.

[45] Alam MT, Merlo ME, Hodgson DA, Wellington EM, Takano E, Breitling R (2010). Metabolic modeling and analysis of the metabolic switch in *Streptomyces coelicolor*. BMC Genomics.

[46] Isiegas C, Parro V, Mellado RP (1999). Streptomyces lividans as a host for the production and secretion of Escherichia coli TEM β-lactamase. Lett Appl Microbiol.

